# Health Service Utilization and Its Determinants among Senior Citizens in the Semiurban Area of Western Nepal: A Cross-Sectional Study

**DOI:** 10.1155/2023/3655259

**Published:** 2023-12-19

**Authors:** Yamuna Chhetri, Dhurba Khatri, Nand Ram Gahatraj

**Affiliations:** ^1^Patan Academy of Health Sciences, Lagankhel, Lalitpur, Nepal; ^2^School of Health and Allied Sciences, Pokhara University, Pokhara, Kaski, Nepal; ^3^Kathmandu Institute of Child Health, Hepaliheight, Kathmandu, Nepal

## Abstract

**Background:**

Senior citizens are usually infected by multiple chronic conditions and other health problems. Health needs and demand for healthcare services increase with age. However, healthcare services and facilities and their utilization are limited, particularly in developing countries.

**Aims:**

To identify the utilization of health services among senior citizens and their contributing factors.

**Methods:**

A cross-sectional analytic study was conducted among 293 senior citizens of the Kushma municipality, Nepal, from June to December 2019. A structured questionnaire was used as a data collection tool using a multistage sampling technique. Face-to-face interviews were conducted to collect data on the interview schedule. Reliability and validity were maintained by applying different strategies and carefully developing tools, pretesting, double entry, and validation. Data entry, management, and analysis were performed using Epi Data and SPSS software. Research ethics were maintained. Descriptive and inferential statistical tests were performed to infer the findings.

**Results:**

Study participants had a mean age (±SD) of 70.08 (±7.6) years and had various preexisting chronic diseases such as hypertension (46%), gastritis (41.9%), arthritis (34.3%), and asthma (28.7%). Only eight out of ten senior citizens had used health services in the past year. Factors such as age, ethnicity, residency, household income, family support, the presence of chronic diseases, and being under medication were found to have statistically significant associations with the utilization of health services among senior citizens with a *p* value less than 0.05 and 95% confidence interval.

**Conclusions:**

A remarkable proportion of older people reported using health services in the last year. However, a substantial proportion did not utilize health services that require further interventions to enable them. Efforts are required to promote the health and well-being of Nepal's growing elderly population, including potential enhancements to rural healthcare infrastructure by policymakers.

## 1. Introduction

Ageing is a natural and inevitable phenomenon that causes gradual changes in physical appearance and mental state and a growing risk of disease. The Senior Citizens Act of Nepal 2063 defines senior citizens as “people who are 60 years and older” [1–3]. The older population is in increasing trend all around the world, and Nepal is no exception [4, 5]. The World Health Organization (WHO) predicted that the proportion of the world's population over 60 years of age would nearly double from 12% to 22% between 2015 and 2050 [1, 6, 7]. The percentage of older people aged 60 and above in the Asia-Pacific region is projected to increase from 8% in 2015 to 18% by 2050 [8]. In 2011, Nepal's census revealed 2.1 million elderly people, making up 8.1% of the population [9]. With the improvement in living standards, educational status, falling fertility and mortality rates, and health facilities, life expectancy contributes to the number of seniors [10, 11].

Ageing is associated with chronic health problems such as cardiovascular disease, cancer, and neurological disorders, which pose a significant challenge to healthcare systems and are associated with higher healthcare costs [12–14]. The utilization of healthcare services among the elderly may be influenced by various personal, social, economic, and environmental factors, and despite the high burden of disease among older people, they tend to use fewer health services than younger adults [1, 12, 15]. The growing elderly population is a global issue that will have a major impact on healthcare policies and programs [10].

The population of senior citizens in Nepal is increasing both in absolute numbers and as a proportion of the total population, resulting in a growing number of elderly people suffering from various problems [5, 16, 17]. However, studies in this group are limited. Healthcare consumption patterns among the elderly are complex, and their determinants may vary depending on factors such as time, socioeconomic status, and geography, which can influence their utilization of healthcare services, and this study deals with that [14, 18].

## 2. Materials and Methods

### 2.1. Study Design

A community-based quantitative cross-sectional analytical study was conducted from June to November 2019. This study was carried out in Kushma, which is the capital of the Parbat district and lies in the western part of Nepal. The total population of Kushma municipality was 40,374, with 18,470 males and 21,904 females [19].

### 2.2. Study Population

All older adults over 60 years residing in Kushma municipalities for the past year were the study population.

### 2.3. Inclusion and Exclusion Criteria

The study's inclusion criteria were senior citizens aged 60 years and older, who had resided in Kushma municipality for at least one year. The exclusion criteria were older adults who had hearing and/or speech-related problems, mental illness, or critical illnesses that limited their ability to provide appropriate responses. Furthermore, if multiple senior citizens in a household met the inclusion criteria, the youngest was excluded from the study.

### 2.4. Sample Size and Sampling

A sample size of 293 was estimated using Cochran's formula for the finite population, assuming a population of 3210 senior citizens residing in Kushma municipality, a proportion of healthcare utilization of 0.7, a 95% confidence interval, and a maximum allowable error of 5%. The samples were selected using a multistage sampling technique. The sampling frame and accompanying data were based on the 2011 Census data. In the Kushma municipality, which has 14 wards, eight wards were selected using a simple random sampling technique with the aid of a random number generator application. The required sample size for each ward was determined using a probability proportional to the number of households in each ward. The households were selected from each ward. To select households, the center of each catchment area was initially reached and a direction was chosen using spinning arrows on a mobile app. Data were collected by visiting the home in the direction indicated by the arrow. If the selected household did not have any eligible participants, the “nearest door” criterion was applied, which meant visiting the household whose front door was neighboring to the selected household's front door. An eligible respondent from each household was surveyed, and if there were multiple eligible participants, the oldest was interviewed.

### 2.5. Data Collection

Before the face-to-face interviews, the tool was pretested on 29 elderly individuals in Beni municipality. After modifications were made based on the pretest, structured questionnaires were used to conduct face-to-face interviews to collect detailed information from participants on their socioeconomic conditions, health status, healthcare service utilization behavior, and satisfaction with health services. Furthermore, participants were asked to present their patient records, such as doctor prescriptions, to confirm their visits to health institutions.

### 2.6. Study Variables

#### 2.6.1. Dependent Variables

Health service utilization was there in the past 12 months.

#### 2.6.2. Independent Variables

Based on Anderson's behavioral model, the factors of predisposing, enabling, and need were included as follows. Predisposing factors included characteristics such as age, sex, ethnicity, religion, marital status, educational status, type of family, residency, and awareness of free healthcare services. Enabling factors included personal monthly income, family monthly income, awareness of insurance, membership in an insurance plan, family support, and the individual responsible for household decision-making. The need factors included perceived health, frequency of health problems, use of medications, difficulties experienced in the past month, and the presence of disabilities. Health service-related factors included the timing of the last healthcare need, type of healthcare facility utilized, time required to reach the facility, mode of transportation used, hospital admissions, duration of the overnight hospital stay, and dependence on healthcare expenses. Satisfaction with health services was measured using an 11-item Likert scale.

### 2.7. Quality Control and Assurance

The tool was developed solely on experts' opinions and an extensive review of the literature. Pretest was performed to enhance the reliability and validity of the tools. The tool was translated into a simple Nepali language, and back translation was applied. Data were entered into the Epi data to help prevent errors. To reduce reporting bias, each participant was informed of the purpose of the study before the investigation.

### 2.8. Data Management and Analysis

The collected raw data was first cleaned, coded, and entered into EPI DATA version 3.1. Subsequently, all the entered data were transferred to the Statistical Package for Social Sciences (SPSS) for further analysis. Descriptive analysis, including frequencies, means, percentiles, and more, was performed. Inferential analysis was also conducted, which included chi-square tests and logistic regression analysis.

### 2.9. Ethics Approval

Before conducting the study, ethical approval was obtained from the Pokhara University, Institutional Review Committee (Ref No. 48/076/077). Permission to conduct the study was also obtained from Kushma municipality. Written informed consent was obtained from each participant after explaining the research objectives, procedures, and confidentiality. The information was kept confidential and only used for the study.

## 3. Results

The mean age of the research participants was 70.08 years with SD 7.618. More than half (52.6%) of the respondents were female. Over three-fifths (63.8%) of respondents were from the upper caste, and most of them followed Hindu religion (91.8%). The majority of the respondents (67.2%) were living with their spouse. In our study, more than half (51.2) of the respondents were illiterate. The majority of study participants were living in joint families (62.5%), residency in semiurban areas (72.7%), knew the benefits package (60.4%), and all the participants were known about the old age allowance (Table 1).

Two-fifths of the study participant's (40.7%) main source of personal income was agriculture. The majority (82.3%) of respondents' monthly income was less or equal to 10,000. Furthermore, more than a fifth (27.4%) of the participant's family income was remittance and more than half (53.4%) of the participants' families had a monthly income of Rs. 50,000 or more. Likewise, more than half (53.9%) of the participants were aware of the insurance program, and only 16.5% had membership in the insurance program. The overwhelming majority of respondents (89.4%) reported that they have any kind of family support for the utilization of health services and most of them (93.1%) get economic support from the family. Likewise, half of themselves (50.9%) were decision-makers for the utilization of health services (Table 2).

More than half of the respondents (54.9%) perceived their health as moderate, and the same percentage of participants (54.94%) faced the health problem 2–5 times in the last year. It was found that more than three-fifths (62.8%) of the participants were on medication. Half of the respondents (50.9%) experienced difficulty doing work or household activities in the past month. Likewise, one-fifth (21.6%) of the participants have some type of disability, and a majority (57.1%) have problems with vision (Table 3).

**Figure 1:**
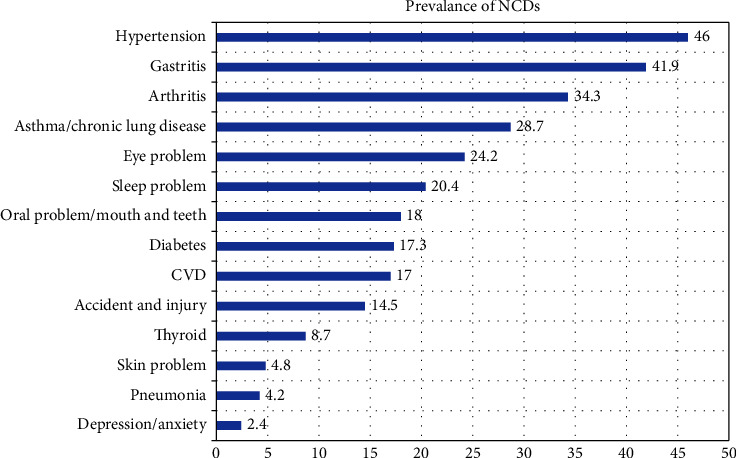
Health problems among study participants.

More than a fifth (81.2%) of the participants utilized health services in the last year. Most (70.7%) of the participants needed health services the last time was within one month, and almost a third (31.1%) of the participants had visited a private hospital. More than a fifth of the participants (62.5%) went to the health facility which was less than 30 minutes, and above half of their (53.8%) mode of transportation was by foot. It was found that 15.5% of the participants were admitted to the hospital in the last year, and more than half (54.1%) stayed in the hospital for 5–15 days. Likewise, more than two-fifth (44.1%) of the participants depend on their son/daughter-in-law for healthcare expenses (Table 4). Almost half (46%) of the total respondents suffered from hypertension followed by gastritis 41.9%, arthritis 34.3%, and asthma 28.7%, while 2.4% suffered from depression/anxiety and so on in the last year (Figure 1).

Male senior citizens had more than two-fold (AOR = 2.082 95% CI = 1.111–3.902) odds of utilizing the health services as compared to female senior citizens. Senior citizens who belong to the privileged ethnic group had more than three times the probability (AOR = 3.129, 95% CI = 1.67–5.84) of utilizing health services as compared to the underprivileged ethnic group. Similarly, senior citizens who lived in the urban area had more than four times the odds (AOR = 4.312, 95% CI = 1.718–10.82) compared to their counterparts, who lived in semiurban areas. Senior citizens whose family income per month was greater than 50,000 have almost five times (AOR = 4.852 95% CI = 1.118–21.08) odds of using health services as compared to their counterparts, whose family income per month was less than 50,000. Senior citizens who had family support had three times the odds (AOR = 3.21 95% CI = 1.45–7.09) of utilizing the health services compared to their counterparts, who did not have family support. Likewise, seniors with chronic disease had almost three times the odds (AOR = 2.90 95% CI = 1.178–7.158) of using health services as compared to those without chronic disease. After adjusting for the effects of other explanatory variables, senior citizens who are under medication have two-fold (AOR = 2.06 95% CI = 1.062–4.006) odds of utilizing health services as compared to their counterparts, who are not under medication (Table 5).

## 4. Discussion

Based on the study, a significant percentage of senior citizens have used healthcare services within the past year. It was determined that the elderly in the Kushma municipality used 81.2% of health services. This result is slightly lower than a study conducted in Butwal which found that 84.4% of senior citizens had visited a health facility during the previous year [4]. The study conducted by Acharya et al., Sanjel et al., and Karmacharya et al. in Pokhara reported lower utilization rates, respectively, of 68% [14], 70% [20], and 77.6% [21] as compared to this study. These variances in health service utilization patterns might be due to the service accessibility, availability of a health workforce, the burden of diseases, the consciousness of people, and the decentralization of health services.

Different health problems such as hypertension, diabetes, arthritis, gastritis, and asthma are common among senior participants in our study and others [14, 20, 22]. In Nepal, the prevalence of diabetes in 2019 was 8.5%, with higher rates in those over 60 years (13.3%) and among males (11%) [23]. Additionally, the prevalence of COPD in the same year was 11.7%, which increased to 21.5% in individuals aged 60 and above [23]. Meanwhile, according to the Nepal Demographic Health Survey, the incidence of hypertension among those aged 60–69 rose from 39.5% in 2016 to 41.6% in 2022, presenting a growing healthcare challenge among the elderly population [24, 25] Being diagnosed with noncommunicable disease shows a significant association with the use of health services, which is consistent with the study by Cotingting et al. in the Philippines [8]. In our study, hypertension was the most common health problem among the senior population, similar to the study of Pokhara conducted by Acharya et al. [14] and Dharan by Adhikari and Rijal [26], but in the study of north India conducted by Sharma et al., musculoskeletal problem was the most common [27]. Predisposing factors such as gender, ethnicity, and residency were associated with health service utilization. Female participants were more likely to use health services than male participants, in agreement with other studies from Nepal [4, 14, 20] and around the world [28–30]. Higher health services used by women may be due to their higher morbidity burden and their protective and preventive attitude towards disease.

Similarly, ethnicity had a significant association with health services, which is similar to other studies where the privileged caste was found to use more health services than the underprivileged caste, such as Dalits and Janjatis [4, 14]. It may be because almost all the underprivileged fall below the poverty line and may have limited access as well as the financial resources to afford healthcare and education [31].

Likewise, this study revealed that elderly people who lived in urban areas were more likely to use healthcare services as compared with elderly people who lived in semiurban and rural areas. This finding was similar to a study conducted in different parts of Nepal [4, 14, 20, 21]. It could be because in rural areas, the availability of healthcare services, human resources, and technology is more limited.

In our study, enabling factors such as household income and family support were statistically associated with health service utilization. More monthly household income makes people more likely to use health services. Acharya et al. [14], Gurung et al. [4], Spaan et al. [32], Babitsch et al. [33], and Ghimire et al. [34] also revealed an association among health service utilization, household income, and distance to the health facilities. It indicates that financial hardship is the greatest barrier to utilizing the health services among senior citizens. However, a study conducted in Pokhara metropolitan city by Karmacharya et al. [21] did not show a significant association between household income and health service utilization. The difference might be that the proportion of participants with high monthly household income was high in Pokhara. It is important to note that the Nepal government provides free social health insurance scheme and free medical care services for senior citizens suffering from Alzheimer and geriatric-related diseases such as Parkinson, heart disease, kidney disease, asthma, and cancer. There is a government provision to establish geriatric ward in the health institution having capacity of more than 100 beds [35]. Likewise, the presence of family support was strongly associated with health service utilization. A previous study from Nigeria also reported that family support was a factor associated with health service utilization among senior citizens [36]. However, healthcare utilization among the elderly is still inadequate. One of the major problems faced by senior citizens was the high level of economic dependence on others, and the use of social security measures among the elderly was very low. Among South Asian countries, Nepal expenses large amount of money in social security. In fiscal year 2022/23, the government of Nepal allocated Rs. 134.01 billion for elderly citizen social security [37]. The government of Nepal introduced the Universal Old Age Allowance Program in FY1994/95, initially providing Rs. 100 per month and subsequently increasing it to Rs. 500, Rs. 1000, and now Rs. 4000 for all elderly individuals aged 68 and above and allocated [38, 39]. However, with increase in life expectancy, the social security of older people is an issue of growing concern.

In our study, health service utilization was not significantly associated with self-perceived health status. However, previous studies have found that self-perceived health status is one of the strongest predictors of health service utilization among seniors [4, 40, 41]. This inconsistency might be due to the difference in perceiving pattern, variation in time of study period, and area as well.

The presence of chronic disease is a strong predictor of health service utilization, which is similar to other studies [4, 20, 28, 40–43]. Likewise, regular medication was another factor associated with health service utilization. These variables indicate that they played a vital role in health service utilization. This finding reveals that the elderly population is more inclined to visit health facilities only when their health condition is alarming like having a chronic disease and being under medication, while the practice of utilizing screening services is low [44].

## 5. Conclusion

Nepal's older adult population is growing quickly, so some action is needed to ensure their health and well-being. A notable proportion of senior citizens did not utilize health services despite having a health problem. Findings indicate that gender, ethnicity, residency, family income, family support, chronic disease, and medication status must be taken into account when understanding and addressing senior health service utilization. Policymakers and healthcare providers should take these factors into account to ensure equitable access to healthcare services for all senior citizens, especially those from marginalized groups and rural areas. Additionally, it is also important to improve the infrastructure and services for healthcare in rural areas to close the health disparity that exists between urban and rural areas, and this will be a foremost task for the public health system in Nepal. Health service utilization among senior citizens needs to be studied in further depth through longitudinal studies.

## Figures and Tables

**Table 1:** Predisposing factors characteristics of participants.

Variables	Frequency (*n*)	Percentage (%)
Age (*n* = 293)		
60–69	156	53.2
70–79	101	34.5
≥80	36	12.3
Mean = 70.08, SD = 7.618, max = 94 years, min = 60 years		
Gender (*n* = 293)		
Female	154	52.6
Male	139	47.4
Ethnicity (*n* = 293)		
Upper caste	187	63.82
Janajati	59	20.13
Dalit	39	13.31
Others	8	2.73
Religion (*n* = 293)		
Hindu	269	91.8
Buddha	18	6.16
Muslim	6	2.04
Marital status (*n* = 293)		
With spouse	197	67.2
Without spouse	96	32.8
Educational status (*n* = 293)		
Illiterate	150	51.2
Able to read and write	85	29.0
Basic education	36	12.3
Secondary	20	6.8
Bachelor and above	2	0.7
Family type (*n* = 293)		
Joint	183	62.5
Nuclear	110	37.5
Residency (*n* = 293)		
Urban	80	27.3
Semiurban	213	72.7
Knowledge about the benefit package		
Yes	177	60.4
No	116	39.6
Know about types of benefit packages^*∗∗*^		
Old age allowance	177	100.0
Concession in treatment	70	39.5
Reservation and concession in transportation	35	19.8
Government-run insurance	25	14.1

^*∗∗*^Multiple choice.

**Table 2:** Enabling characteristics of the participant.

Variables	Frequency (*n*)	Percentage (%)
Source of personal income (*n* = 290)		
Agriculture	118	40.7
Old age allowance	93	32.1
Pension	38	13.1
Business	20	6.9
Others	21	7.2
Participant income per month (*n* = 293)		
Less or equal to 10000	241	82.3
Above 10000	52	17.7
Source of family income (*n* = 277)		
Remittance	76	27.4
Business	67	24.2
Agriculture	56	20.2
Service-related	53	19.1
Others	25	9.1
The family income per month (*n* = 253)		
Less than 13450	30	11.9
13450–50000	88	34.8
50000 and above	135	53.4
Awareness of insurance (*n* = 293)		
Yes	135	53.9
No	158	46.1
Membership of insurance (*n* = 158)		
Yes	26	16.5
No	132	83.5
Family support (*n* = 293)		
Yes	262	89.4
No	31	10.6
Type of family support (*n* = 262)^*∗∗*^		
Economic	244	93.1
Physical	151	57.6
Emotional	128	48.9
Decision taker (*n* = 293)		
Self	149	50.9
Son/daughter-in-law	103	35.2
Partner	31	10.6
Daughter/son-in-law	10	3.4

^*∗∗*^Multiple responses.

**Table 3:** Characteristics related to the need factor of the participant.

Variables	Frequency (*n*)	Percentage (%)
Perceived health (*n* = 293)		
Good	70	23.9
Medium	161	54.9
Bad	62	21.2
Times of health problem faced (*n* = 293)		
1 time	37	12.62
2–5 times	161	54.94
5 or more than 5	95	32.42
Under medication (*n* = 293)		
Yes	184	62.8
No	109	37.2
Difficulty experienced in the last month (*n* = 293)		
Yes	149	50.9
No	144	49.1
Presence of disability (*n* = 293)		
Yes	63	21.5
No	230	78.5
Types of disability (*n* = 63)^*∗∗*^		
Vision problem	36	57.1
Hearing problem	29	46.0
Physical disability	15	23.8
Speech impediment	2	3.2

^*∗∗*^Multiple responses.

**Table 4:** Health services use indicators.

Variables	Frequency (*n*)	Percentage (%)
Utilized health services in the last one year (*n* = 293)		
Yes	238	81.2
No	55	18.8
Last time needed health service (*n* = 290)		
Less than 1 month	205	70.7
More than 1 month	85	29.3
Types of health facility visited (*n* = 238)		
Private hospital	74	31.1
Government hospital	70	29.4
PHC/health post	59	24.8
Pharmacy	28	11.8
Ayurvedic	7	2.9
Time to reach the health facility (*n* = 293)		
<30 min	183	62.5
>30 min	110	37.5
Mode of transportation (*n* = 238)		
By foot	128	53.8
Bus	77	32.4
Taxi	24	10.1
Private vehicle	9	3.8
Admitted in hospital (*n* = 238)		
Yes	37	15.5
No	201	84.5
Duration of overnight hospital stay (*n* = 37)		
1–5 days	15	40.5
5–15 days	20	54.1
>15 days	2	5.4
Depend on healthcare expenses (*n* = 238)		
Son/daughter-in-law	105	44.1
Self	100	42
Partner	24	10.1

**Table 5:** Factor association with health service utilization.

	Variables	Utilized health services		*P* value	COR (95% CI)	AOR (95% CI)	*P* value
		Yes (238)	No (55)				
Predisposing factors	Gender						
	Male	105 (75.5)	34 (24.5)	0.018^*∗*^	2.05 (1.124–3.741)	2.082 (1.111–3.902)	0.022^*∗*^
	Female	133 (86.4)	21 (13.6)		Ref		
	Ethnicity						
	Upper caste	163 (87.2)	24 (12.8)	0.001^*∗*^	2.807 (1.54–5.11)	3.129 (1.67–5.84)	<0.001^*∗*^
	Nonupper caste	75 (70.8)	31.29.2		Ref		
	Residency						
	Urban	74 (92.5)	6 (7.5)	0.002^*∗*^	3.68 (1.51–8.98)	4.312 (1.718–10.82)	0.002^*∗*^
	Semiurban	164 (77)	49 (23)		Ref		

Enabling factors	Personal income						
	≤10000	189 (78.4)	52 (21.6)	0.015^*∗*^	Ref	Ref	0.103
	>10000	49 (94.2)	3 (5.8)		0.223 (0.067–0.743)	2.279 (0.812–9.62)	
	Family income per month						
	≤50000	187 (77.9)	53 (22.1)	0.002^*∗*^	Ref	Ref	0.035^*∗*^
	>50000	51 (96.2)	2 (2.8)		0.138 (0.033–0.587)	4.852 (1.118–21.08)	
	Family support						
	Yes	221 (84.4)	41 (15.6)	<0.001^*∗*^	4.44 (2.03–9.70)	3.21 (1.45–7.09)	0.004^*∗*^
	No	17 (54.8)	14 (45.2)		Ref		

Need factors	Self-perceived health						
	Medium/poor	189 (84.8)	34 (15.2)	0.006^*∗*^	2.382 (1.271–4.465)	0.861 (0.393–1.883)	0.707
	Good	49 (70)	21 (30)		Ref		
	Presence of chronic disease						
	Yes	220 (84.3)	41 (15.7)	<0.001^*∗*^	4.173 (1.92–9.049)	2.90 (1.178–7.158)	0.021^*∗*^
	No	18 (56.2)	14 (43.8)		Ref		
	Under medication						
	Yes	160 (87)	24 (13)	0.001^*∗*^	2.65 (1.46–4.82)	2.04 (1.053–3.961)	0.033^*∗*^
	No	78 (71.6)	31 (28.4)		Ref		

^*∗*^ *p* value significant at 0.05.

## Data Availability

The data used to support the findings of this study can be obtained from the corresponding author upon reasonable request.
